# Review of software for space-time disease surveillance

**DOI:** 10.1186/1476-072X-9-16

**Published:** 2010-03-12

**Authors:** Colin Robertson, Trisalyn A Nelson

**Affiliations:** 1 Spatial Pattern Analysis & Research (SPAR) Laboratory, Dept of Geography, University of Victoria, PO Box 3060, Victoria, BC V8W 3R4, Canada

## Abstract

Disease surveillance makes use of information technology at almost every stage of the process, from data collection and collation, through to analysis and dissemination. Automated data collection systems enable near-real time analysis of incoming data. This context places a heavy burden on software used for space-time surveillance. In this paper, we review software programs capable of space-time disease surveillance analysis, and outline some of their salient features, shortcomings, and usability. Programs with space-time methods were selected for inclusion, limiting our review to ClusterSeer, SaTScan, GeoSurveillance and the Surveillance package for R. We structure the review around stages of analysis: preprocessing, analysis, technical issues, and output. Simulated data were used to review each of the software packages. SaTScan was found to be the best equipped package for use in an automated surveillance system. ClusterSeer is more suited to data exploration, and learning about the different methods of statistical surveillance.

## Introduction

Disease surveillance is an ongoing process of information gathering, organizing, analyzing, interpreting, and communicating. It is the principal means by which public health information is generated and disseminated, informing policy, research, and response measures. For outbreaks of infectious disease, timely information on the spread of cases in space and time can facilitate action by public health officials [e.g., [1]]. For chronic and endemic diseases, monitoring space-time trends in disease occurrence can highlight changing patterns in risk and help identify new risk factors [e.g., [2]]. Analysis of spatial-temporal patterns in public health data is an increasingly common task for public health analysts as more surveillance data become available. Surveillance datasets are often massive in size and complexity, and the availability and quality of software capable of analyzing space-time disease surveillance data on an ongoing basis is integral to practical surveillance [3-5]. Geographic information systems (GIS) used for disease mapping can visualize the spatial variation in disease risk. However, statistical methods are often required to detect changes in the underlying disease process. GIS are also poorly equipped to handle temporal data [6].

In Fall of 2008, a workshop on training priorities in the use of GIS in health research conducted in Victoria, British Columbia, polled 78 researchers, graduate students, faculty, and others working in health and GIS regarding barriers to the use of space-time disease surveillance [7]. Training and software availability were cited as the primary barriers to the uptake of space-time disease surveillance. Currently, statistical methods for space-time disease surveillance are not included in most conventional GIS or statistical software. These methods are available in specialist cluster analysis software such as ClusterSeer http://www.terraseer.com, or as extensions to general statistical analysis software packages (e.g., R, S-Plus). Our goal is to provide researchers and public health analysts with a review and demonstration of software packages for space-time disease surveillance. We aim to facilitate expanded use of these methods by providing a means to quickly determine the software options and to identify the ways in which programs differ. We limit our scope to methods that use both space and time, rather than purely temporal or spatial analysis.

This paper is organized as follows. First, we briefly review basic classes of methods for space-time disease surveillance in the background section. Readers familiar with these methods may wish to skip ahead. Second, in the methods section we outline how we selected software to review, the review methodology and datasets used to demonstrate software features. Third, we present the results of our review. Finally, we conclude with some guidelines for the use of these software packages for public health researchers and analysts.

## Background

Statistical approaches to disease surveillance have been the subject of a number of texts and review papers [8-10]. A key factor in the selection of methods of analysis is the objective of surveillance, such as outbreak detection, trend monitoring, case detection, or situational awareness. Additional contextual factors are also important to consider such as scale and scope of the system, disease characteristics, and technical considerations [11]. Methods can be broadly categorized as either statistical tests or model-based approaches. Statistical tests are the dominant class of approaches used for outbreak detection. The aim of most methods is to test a subset of data, defined by spatial and temporal constraints (i.e., a window or kernel), against an expected rate of disease occurrence over the study area as a whole. Methods differ with respect to how the window that defines each subset is constructed, how statistical significance is determined, and how the baseline expectation varies over space and time.

The most widely used testing methods are cumulative sum (cusum) methods and scan statistics. Briefly, cusum approaches keep a running sum of deviations from the expected value, and once the cumulative deviation reaches some threshold, an alarm is triggered. For space-time applications, individual cumulative sums for each area under surveillance are monitored and can be adjusted for spatial relationships [12]. Depending on the statistic being monitored in the cusum, different surveillance objectives can be addressed. For example, a measure of spatial pattern monitored in a cusum framework can be sensitive to slight changes in spatial pattern which may signal a shift in dynamics of an endemic disease [e.g., [13]]. Scan statistics are used mostly in outbreak detection contexts. Here, circular search windows of varying radii scan a map of disease and test if the number of cases within the search area is unexpectedly high. In the space-time scan statistic [14,15], the search area is extended to a cylinder where the height of the cylinder is defined by time periods of varying lengths. The mostly likely cluster is assessed using monte carlo simulations.

Modeling approaches are used mostly for adjusting the expected number of cases (i.e., denominator) of disease. Disease incidence varies spatially with population and known risk factors. Disease mapping models aim to estimate the true *relative risk *across the study area by incorporating the spatial variation in these risk factors. The standardized mortality ratio (SMR) is the crudest measure of risk, computed as the observed cases divided by the expected in each area. The SMR is often of limited use in surveillance because it can fluctuate widely for rare diseases or in rural areas where populations are small. Further, abrupt (i.e., unrealistic) changes at the boundaries of areal units are sometimes observed. Models allow both covariate effects to be estimated, and for sparsely populated areas to have their expected values adjusted towards the mean (i.e., borrow strength). When used in surveillance applications, models confer these same advantages. Disease surveillance models have been either space-time Bayesian models [e.g., [16]] or generalized linear mixed models [e.g., [17]]. Modeling approaches are complementary to other methods as tests are still required to determine how well the most recently observed data fit with the model [18]. Adjustments can also be such that models can be refit over time to adjust to long-term changes in disease occurrence or surveillance effort/efficacy (e.g., improved diagnostic tests), and parameters can be included to model spatial relationships and seasonal and day of the week effects, common features of some types of disease surveillance data.

In addition to testing and modeling methods, new computation-based tools are also being developed for surveillance. These approaches tend to be in either experimental and/or theoretical stages or algorithms designed for specific surveillance systems. Some hybrid approaches include networks [19], simulation-based methods [20], and space-time hidden markov models [21]. While many of these new approaches appear promising, most are not yet available in software.

## Methods

### Inclusion Criteria

Software programs were included for review based on two criteria: the program had methods that handled both space and time, and methods were built-in to the software (i.e., not requiring programming). This criteria constrained our review to four software packages (SaTScan 8.0, ClusterSeer 2.3, GeoSurveillance 1.1, Surveillance package 1.1-2 for R). Comprehensive disease surveillance systems (also sometimes called health information systems) software, that include data collection and processing routines, database components, and system-specific analysis and visualization modules were excluded (e.g., RODS [22], AEGIS [23]). These systems are large in scale and generally implemented at an enterprise level; they are not readily accessible to researchers/analysts. Research tools based purely on programming (e.g., WinBUGS; MatLab) were also excluded. Details of the software packages included in the review are outlined in Table 1.

**Table 1:** List of software packages for review of space-time disease surveillance software

Software Package	Source	Reference	Description
SaTScan 8.0	http://www.satscan.org	Kulldorff and Information Management Services 2009 [38]	Cluster detection software with several spatial, temporal and space-time scan statistics.

ClusterSeer 2.3	http://www.terraseer.com/	Jacquez et al. 2002 [39]	Cluster analysis software includes many methods for spatial, temporal, and space-time analysis.

GeoSurveillance 1.1	http://www.acsu.buffalo.edu/~rogerson/geosurv.htm	Yamada et al. 2009 [40]	Implementation of cumulative sum surveillance statistics.

Surveillance package 1.1-2	http://cran.r-project.org/web/packages/surveillance/index.html	Höhle 2007 [41]	Package for statistical surveillance includes test-based and model-based methods.

### Reviewing Framework

Software programs were reviewed for broad steps of typical data analysis: preprocessing, analysis (methods and technical issues), and output (Table 2). Preprocessing is required to transform data into the appropriate structure for a particular software package. In many scenarios, health event data are collected at an address level, which needs to be compared to population estimates, available usually as polygon census data [24]. Our assessment of data preprocessing requirements reflected typical data by considering both point event case data, and polygonal administrative units. For each software package, we assessed the data formatting steps required to perform an analysis.

**Table 2:** Criteria and review approach for review of space-time disease surveillance software

Criteria	Review
Data preprocessing	Number of steps involved to process a point event (cases) shapefile and a polygon census shapefile (population)

Methods	Description of methods offered by each program

Technical issues	Speed of computation, system stability, automation, operating requirements

Analysis output	Output options (graphs, maps, reporting)

User facility	Qualitative assessment rated on scale of 1 - 5 on each of:
	• Ease of learning
	• Use
	• Set up
	• Documentation/Help

The second step is conducting the analysis and we briefly describe methods and analysis options for each software package. We highlight technical issues and potential problems or requirements such as stability, speed of computation, and required operating systems. The final step is outputting results and we overview output options available in each package. In addition, we qualitatively assess user facility based on our experience operating the software with test datasets. It should be noted that we do not discuss parameterization of different methods. This is a major issue in practical surveillance, suited to a review and comparison of surveillance methods themselves.

### Datasets

Data were simulated to model a syndromic surveillance system monitoring calls to a health hotline in the Greater Vancouver Area. For simplicity, we refer to each simulated call as a case. Cases were simulated over one year from January 1^st ^to December 31^st^. Cases were aggregated to census dissemination areas (DA) and were spatially allocated proportional to the population in each census DA. The total population in all DAs was 578,642, and total cases were 4303, giving an annual incidence of 743.64 cases per 100,000. This level of incidence is similar to what might be expected for the total volume of calls made to a telephone health hotline in a major Canadian city [25].

**Figure 1:**
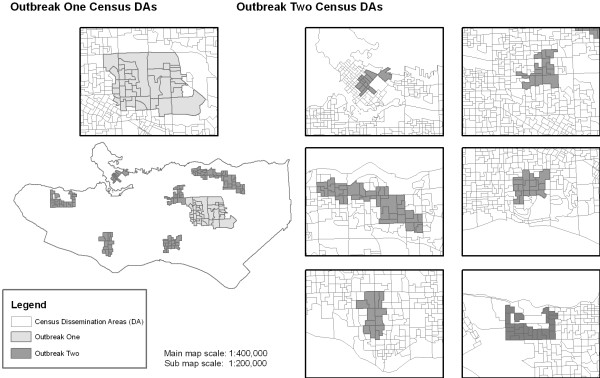
**Outbreaks simulated to review software packages for space-time disease surveillance (Outbreak one - light grey; Outbreak two - dark grey)**. Outbreak one consisted of one large compact cluster. Outbreak two was composed of several clusters occurring at different times throughout the region.

Outbreaks were inserted into baseline data to indicate signals of a spike in calls which, in a sydnromic surveillance setting, indicate a signal of an unusual health event. Two outbreak scenarios were simulated in separate datasets. In outbreak one, a simulated outbreak started on March 4^th ^and lasted until June 5^th^, with 148 cases occurring over 10 sq km, covering 33 geographically adjacent census DAs (light grey cluster, Figure 1). Outbreak cases were allocated proportional to census DA population. In outbreak two, 6 spatial clusters constituting a total 501 cases occurred over an area of 16 km^2^, covering a total of 104 census DAs (dark grey cluster, Figure 1). The number of cases in clusters ranged from 51 to 140, and cases occurred over the full year. Data were stored in Environmental Systems Research Institute (ESRI) shapefile format, a standard spatial data format which can represent data as points, polygons, or lines.

### Review of Programs

#### Data preprocessing

The steps involved in preprocessing the test data for analysis in each software program are outlined in Table 3. SaTScan requires data to be input as three separate files to run the appropriate analysis for this data (retrospective space-time scan, Poisson model) where one file stores the spatial locations (geo file), another file stores the cases (case file), and a third stores the population of each area (population file). All SaTScan files are text-based, and an import tool is provided for importing common data formats (e.g., CSV, DBF). SaTScan also provides the functionality to aggregate the data temporally into years, months, or days. Thus, data can be input at the finest temporal resolution. This functionality turned out to be a key advantage over other programs as it limited the amount of data restructuring required when trying different analysis parameters.

**Table 3:** Data preprocessing steps for each software package to perform a space-time analysis starting with daily data as point events in an ESRI point shapefile and a polygon shapefile of census dissemination area boundaries

Software	Type of Analysis	Required Data Structure	Data Preprocessing Steps
SaTScan	Space-time cluster scan with Poisson model	• Case file with number of cases, date, and DA id • Population file with population, date, and DA id • Coordinates file with DA id, centroid X and Y coordinates	• Associate DA identifier with each point event • Calculate DA centroid coordinates

ClusterSeer	Space-time cluster scan with Poisson model	• One table with population • One table with counts of cases for each location and date during study period	• Associate DA identifier with each point event • Calculate week numbers • Aggregate cases by week for each DA (zero counts included)

GeoSurveillance	Univariate cusum on individual DAs	• DA shapefile with counts of number of cases for each time period named and ordered sequentially in the table	• Calculate week numbers • Split point events into unique shapefiles for each week • Count number of events in each DA by week (zero counts included) • Calculate weekly counts as new fields

R-Surveillance	Univariate cusum on individual DAs	• Matrix of counts of cases with spatial locations as columns and time periods as rows	• Calculate week numbers • Split point events into unique shapefiles for each week • Count number of events in each DA by week (zero counts included) • Calculate weekly counts as new fields • Read table into R as matrix and transpose

ClusterSeer requires unique records for every space-time unit under surveillance. Running a daily space-time scan statistic for our simulated data would require a dataset with four columns (location, date, cases, population) and 478,515 records (365 days × 1311 census DAs). Additionally, all areas need a record for every time period. Generating the necessary table required use of specialized data restructuring functions in R statistical software (reshape package). Data were aggregated to counts of cases by week. (52 weeks × 1311 census DAs) giving a table with 68,172 records. For weeks where DAs had no cases, zero counts had to be inserted.

Preparing data for analysis in GeoSurveillance required aggregation temporally and spatially. Counts of cases were required to be attributes of the polygon shapefile (or text file), and fields were required to be named in sequential order. This process was automated by custom programming in ArcGIS which performed spatial joins and added new fields to the attribute table. This was an extensive process to get the data in the proper format for analysis, and similar to ClusterSeer, GeoSurveillance does not allow flexibility in the level of temporal aggregation. ClusterSeer and GeoSurveillance can both read in polygon shapefiles and automatically calculate centroid coordinates.

For analysis with the Surveillance package in R, data were required to be in a matrix with temporal observations as rows and spatial units as columns, giving a 365 × 1311 matrix for daily analysis and 52 × 1311 for weekly analysis. All of the programs except SaTScan had inflexible data input requirements, specifically for temporal aggregation of cases. None of the software programs could input the two shapefiles (points and polygons) without any data preprocessing. This was surprising as previous experience and a review of SaTScan [26] suggested cumbersome input format as a major limitation of SaTScan.

#### Methods

The programs reviewed here are of two types: specialized implementation of a specific class of surveillance algorithms (SaTScan, GeoSurveillance) and full suite surveillance/space-time analysis packages that implement multiple methods (ClusterSeer, R-surveillance). SaTScan offers a number of scan statistics such as spatial [27], temporal [28], and space-time versions [14,15], as well as retrospective and prospective (clusters must be current) modes. Different data types can be accommodated by the many probability models including Poisson, Bernoulli, space-time permutation, multinomial, ordinal, exponential, and normal. The circular search area used in the classical scan statistic can also be altered to search using an ellipse, or along user-defined connections of spatial units. GeoSurveillance implements the cusum approach to surveillance [e.g., [13]]. The retrospective mode does global spatial analysis only (i.e., reports one cusum test statistic for the map), while the prospective mode does univariate parallel surveillance with the cusum statistic. The multivariate cusum is not yet implemented in GeoSurveillance.

ClusterSeer had the widest range of space-time methods implemented. Those particularly suited to disease surveillance included space-time scanning [14], a cusum approach similar to that in GeoSurveillance [13], and tests for space-time interaction [29-31]. This makes ClusterSeer a useful tool for exploring disease surveillance data. Once data is formatted for use in ClusterSeer, a variety of methods can be used to examine the data. The R-Surveillance package contains a number of algorithms such as the Farrington et al. (1996) method [32], Poisson cusum [33], and the two-component negative binomial model in Held et al. [34]. The algorithms in the surveillance package are mostly model-based and non-spatial, though some space-time surveillance applications can be treated as a multivariate time series problem.

#### Technical Issues

Technical issues encountered in running the software programs varied considerably. SaTScan was capable of running the space-time scan statistic in retrospective mode on daily case data. ClusterSeer was not run on daily data. Initially, memory requirements were a serious limitation of undertaking analysis in ClusterSeer with both test datasets; however an updated version (2.3.22.0) was obtained to complete the analysis on weekly data. The analysis took longer to run than on SaTScan with daily data, though results were very similar. GeoSurveillance ran the univariate cusum in parallel on each of the 1311 census DAs. The analysis ran well on weekly data, however the linked display between the maximum cusum and the map was very slow. The cusum methods were also used for our analysis in R-Surveillance. The time taken to run the analysis on the weekly data was similar to that of GeoSurveillance and results were also similar.

R-Surveillance is the only package that runs on windows, mac and linux operating systems. Currently, SaTScan has versions for windows and linux, and a mac version is in development. Both ClusterSeer and GeoSurveillance run only on the windows operating system. All analyses were run on a Pentium 4 PC with 3.00 GHz processor and 2 GB of RAM running the Windows XP operating system. SaTScan completed analysis in the shortest time compared to all other programs.

#### Data Output

Output options in SaTScan are limited to text file and database file output. Database files can be linked back to the input shapefile in a GIS for further examination of clusters, however no data exploration functionality is available in SaTScan itself. In GeoSurveillance results of an analysis can be written to text file which can be easily manipulated in other software. GeoSurveillance provides a basic map interface linked to a list of cusum scores. A cusum chart is also displayed showing the temporal pattern of cusum scores for the study area as a whole and individual units.

ClusterSeer has advanced data output facility such as mapping and graphing which can be exported as images. Results can also be exported with the data to new files for further examination inside statistical or GIS software. The Surveillance package has access to extensive visualization and exporting functions available in the R environment. The objects specific to the Surveillance package also have default methods for creating plots. This of course requires familiarity with the R programming language.

#### User Facility: Ease of Learning, Ease of Use, Help & Documentation

Usability is an important part of software as public health organizations have limited resources available for technical training. Our review of user facility is presented in Table 4. ClusterSeer includes an extensive help menu explaining the parameters and required data for all of the methods. The help system also includes tutorials and example datasets that work through many of the methods. This is an important resource for learning methods of spatial and space-time analysis. The graphical user interface (GUI) of ClusterSeer makes learning and use straightforward. SaTScan is also a GUI-based system composed of three main screens: input, analysis, and output. The help menu in SaTScan is extensive with descriptions of the scan statistic methodology, explanations of parameters and data input and output options, sample datasets, and references for further reading. GeoSurveillance has two basic modes which are run from menus of a simple GUI. The program is easy to use after data has been formatted properly (as described above). Currently there is no help built into the system itself. The menus are described in a separate word document. A tutorial and sample datasets are also provided. Having these outside of the program itself makes navigating the documentation cumbersome. R-Surveillance is an R package and as such has help in the R package format, which can be called directly from R. This includes descriptions of parameters and values for all of the implemented functions in the package. Basic examples are given, although detailed descriptions of the statistical methods is lacking. Users should be familiar with using R packages and the background statistical methodology before using the surveillance package.

**Table 4:** Comparative review of software packages for space-time disease surveillance: User Facility

Software	Learning	Use	Set Up	Help/Documentation	Comments
SaTScan	4	5	5	4	Requires knowledge of scan statistics. Basic analysis is straightforward though many advanced options available. Well referenced methodology in the user guide.

ClusterSeer	5	5	3	5	Excellent documentation and learning resources for the many different methods. Data format requirements can be cumbersome.

GeoSurveillance	3	3	3	3	Data structure is peculiar, though the basic user interface is straightforward. Documentation not integrated within the menu itself.

R - Surveillance	1	3	5	2	Command driven system requires knowledge of R language. Examples are easy to replicate. Very easy to install within R. Documentation is not extensive.

## Conclusions

With the advent of electronic medical records, syndromic data sources, and low-cost location sensors, disease data are increasingly encoded with both spatial and temporal information. These new data sources represent an opportunity for greater understanding of disease distributions, risk factors, and changes to population health over time and space. While analysis of surveillance data represents an expanding opportunity for public health practice and research, these new datasets, methods, and software also bring challenges. There are inherent problems in using traditional statistics for hypothesis testing, or applying simple GIS visualization, to these data sources. As is evidenced by the growing literature on statistical surveillance of disease data [9], methods need to be specifically suited to these data. In addition to statistical methods however, computer software is now essential for the analysis of surveillance data.

The four software programs reviewed in this paper provide functionality for different kinds of analysis and serve different purposes. Based on our review, SaTScan is the most developed and robust software package for implemention in an automated cluster detection system. However, SaTScan only implements scan statistic methods, so those wishing to explore modeling-based approaches may want to use the Surveillance package. Additionally, examining the results in detail requires other software for graphing and mapping. Reasons for taking a modeling approach include making refined estimates of expected rates based on modeled covariate effects, adjusting for spatial heterogeneity in disease rate, and smoothing relative risks. The Surveillance package implements models, but currently has very limited capability for true space-time surveillance. The large number of temporal methods make it a useful environment for exploring surveillance data, in addition to the advantages afforded by being able to integrate with other R packages. As a command-based system, it also is easy to automate and integrate with data processing scripts. The learning curve for R is quite steep, and those requiring a GUI-based system to explore surveillance data would be better served by ClusterSeer. The extensive documentation and many purely spatial and temporal methods, in addition to space-time methods, makes it a convenient tool for initial data exploration. There is also a range of output options in ClusterSeer. ClusterSeer may be more suitable for exploratory studies than as part of an ongoing, automated cluster detection system because there is limited capacity for automated surveillance. ClusterSeer project files can be set to run automatically, though because they are binary files they cannot be automatically configured to increment parameters (e.g., study period). Finally, though methods (and software) have been classified as testing or model-based approaches, it is important to note that these approaches are complimentary rather than opposing [18]. For example, one approach is to develop a model of the expected risk of disease using the Surveilllance package, and use the estimated smoothed rates as the expected values in a SaTScan analysis.

All of the programs reviewed in this paper were applications installed on a local computer. While this is the architecture of most computer software applications, new developments in computing are taking advantage of the internet to perform ongoing, high-powered computing tasks [35]. Online delivery of analytic services (such as cluster analysis) allows software to be centralized on one server, and accessible from anywhere with an internet connection. In the context of disease surveillance, this could facilitate standardization of analysis among different regional health authorities, increase transparency of analysis, and offer significant improvements in costs and performance. Initial steps towards web-based surveillance analysis are underway, with a web-based version of ClusterSeer https://www.clusterseer.com currently in development, RWeb [36], a web-based interface to a server instance of R, as well as a newer project called rapache [37], which integrates R into the popular Apache web server. These developments hold considerable promise for the development of future surveillance systems.

The threat of emerging diseases and the growing burden of chronic diseases requires integrated approaches to surveillance. Analysis of disease trends in space-time provides context which can be linked to possible risk factors in a research environment, flag unusual events in an automated surveillance system, and provide epidemiologists with current information during an outbreak. Well-studied and understood methods are required to ensure appropriate use and transparent and reproducible results. The literature on statistical surveillance is extensive and provides this basis, yet software implementations are far from standardized. As space-time surveillance statistical methods mature further, software is also surely to improve. The open-source environments, such as R, may be the optimal venue for future development of surveillance software as they afford easy integration with many statistical and mapping packages, and being open-source, the underlying code can be viewed and modified easily. However data structure remains a major issue when handling space-time data, especially when data has to be moved between different software packages. Standardized space-time data classes in R or another open-source environment may be a fruitful area of development.

## Competing interests

The authors declare that they have no competing interests.

## Authors' contributions

CR and TN conceived of the review, and participated in its design. CR performed all software analysis and data processing. All authors read and approved the final manuscript.
